# Metastasis Patterns and Prognosis of Elderly Patients With Esophageal Adenocarcinoma in Stage IVB: A Population-Based Study

**DOI:** 10.3389/fonc.2021.625720

**Published:** 2021-05-28

**Authors:** Guanghao Qiu, Hanlu Zhang, Fuqiang Wang, Yu Zheng, Zihao Wang, Yun Wang

**Affiliations:** Department of Thoracic Surgery, West China Hospital, Sichuan University, Chengdu, China

**Keywords:** esophageal adenocarcinoma, elderly patients, treatment, metastasis, prognosis, chemotherapy, radiotherapy, surgery

## Abstract

**Background:**

Esophageal adenocarcinoma (EAC) is the most common kind of esophageal cancer. Age at diagnosis of advanced EAC is greater. Studies about practice patterns for elderly EAC patients with distant metastasis (DM) in stage IVB are limited. This retrospective, population-based study was conducted using data from the Surveillance, Epidemiology, and End Results (SEER) to evaluate 855 elderly EAC patients with DM in stage IVB from 2010 to 2015.

**Methods:**

855 elderly EAC patients with DM in stage IVB between 2010 and 2015 were included in this study. Univariate and multivariate Cox-regression and Kaplan-Meier analyses were used to assess prognosis. These patients were classified to bone-only, brain-only, lung-only, liver-only, and multiple (patients with two or more organs in metastasis)-site group according to the site of metastasis. Overall survival (OS), cancer-specific survival (CSS), median survival time (MST), and survival rate (SR) were evaluated to analyze the survival outcomes.

**Results:**

The most common metastasis site was the liver among the single-organ metastasis population, followed by lung, bone, and brain. Compared with the bone-only group, the multiple-site group was associated with worst OS (HR: 1.037, 95% CI: 0.811–1.327, *p* = 0.770) and CSS (HR: 1.052, 95% CI: 0.816–1.357, *p* = 0.695). The multiple-site group also had the lowest MST in the population (MST: 2 months in OS and 3 months in CSS) and SR (6-month SR: 27.1% in OS, 29.9% in CSS, 1-year SR: 10.7% in OS, 12.0% in CSS, 3-year SR: 2.5% in OS, 2.8% in CSS). Compared to untreated patients (N) in the total population, other patients who were treated with surgery (S), radiotherapy (R), and chemotherapy (C) are beneficial for the prognosis (OS and CSS: *p* < 0.001).

**Conclusion:**

This population-based study was conducted to ascertain metastasis patterns and survival outcomes of EAC patients with DM in stage IVB. Elderly patients with multiple-site metastasis exhibited the worst OS and CSS among all the populations, and patients with bone-only metastasis had the worst OS and CSS among single-organ metastasis populations. Active treatment is beneficial for elderly EAC patients with DM in stage IVB, especially chemotherapy. This study also shows that more than one third of the patients had not received any therapy.

## Introduction

Esophageal cancer (EC) is the seventh leading cause of cancer-related mortality in America, the sixth leading cause of cancer-related mortality worldwide, and its incidence continues to increase (1, 2). According to the incidence data of EC extracted from 12 countries, the number of EAC cases is expected to increase rapidly from 2005 to 2030, while the incidence of esophageal squamous cell carcinoma (ESCC) will continue to decline (3). The GBD 2017 Esophageal Cancer Collaborators estimated that there were approximately 473,000 new cases of EC all over the world in 2017 (age-standardized incidence of EC was 5.9 per 100,000 population) and 436,000 deaths (age-standardized mortality of EC was 5.5 per 100,000 population) (4).

With the extension of human life expectancy, the number of elderly EC patients will increase significantly in the future. According to the website of Cancer Research UK, more than 57% of EC patients were over 70 years old (5). Yuan Zeng et al. showed that, among people suffering from EC, compared with patients under 70 years of age, patients over 70 had distinctive clinical characteristics and inferior survival rate (6). There were also many studies of EC patients over 70, which proved that age alone was not a contraindication for surgery and neo-adjuvant chemo-radiotherapy made sense in treating EC patients over 70 (7, 8); however, there are no relevant studies on elderly EC patients with distant metastasis (DM) in stage IVB. Many causes can lead to death in patients with EC, including nutritional disorders, cachexia, local invasion of large blood vessels, *etc.*, but clinical studies indicated that DM was the most common cause of death (9): because the prognosis of patients with EC is poor and more than 50% of patients have lymph node or distant organ metastasis at first diagnosis, it is important to understand the metastasis pattern and prognosis of elderly EC patients with DM (9, 10).

To analyze DM patterns and prognosis of different metastasis groups in a large cohort of the elderly EAC population, we undertook this study by using the SEER database. As many studies on elderly patients use 70 years as the age threshold to define the elderly cohort (11–16), based on site of metastasis, patients were divided into bone-only group, brain-only group, lung-only group, liver-only group, and a multiple-site group. We compared both OS and CSS of these groups with metastasis to different single organs or a combination of multiple organs. Other clinicopathological parameters such as gender, race, grade, T stage, N stage, site of EAC, and treatment were included.

## Materials and Methods

### Data Collection

**Figure 1:**
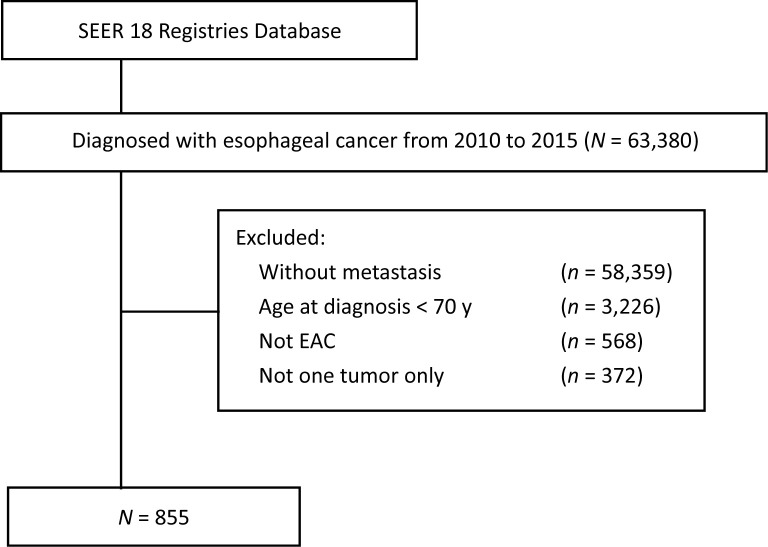
Flowchart of patient selection from SEER database (2010–2015). SEER, surveillance, epidemiology and end results; EAC, esophageal adenocarcinoma.

The SEER 18-Registry custom data (with additional treatment fields, 1975 to 2016, data set submitted in November 2018) of the NCI were analyzed. The eligibility criteria included the following: (1) age ≥ 70 years; (2) Histology codes 8140-8211, 8255-8490, and 8574 were used to define EAC; (3) The primary site codes C15.0 (cervical esophagus) and C15.3 (upper third of the esophagus), C15.4 (middle third of the esophagus), C15.2 (abdominal esophagus), and C15.5 (lower third of the esophagus) were defined as the upper esophagus, middle esophagus, and lower esophagus, respectively. (4) Patients in stage IVB (since the SEER program included data pertaining to four site-specific distant metastases. Exclusion criteria included the following: (1) the values “histologically confirmed positive” were selected to exclude those without histological diagnosis; (2) the values “complete dates are available and there are 0 days of survival” and “complete dates are available and there are more than 0 days of survival” were selected to exclude those without survival data; (3) the values “active follow-up” were selected to exclude those without follow-up data. The flowchart demonstrates the patient selection from SEER database (**Figure 1**).

### Statistical Analysis

Based on site of metastasis, patients were divided into bone-only, brain-only, lung-only, liver-only, and a multiple-site group. Clinical and demographic characteristics were compared for patients of different metastasis groups using Pearson’s chi-squared test statistics for categorical variables. We did the Kaplan-Meier survival curves by the log-rank test, which was used to analyze the differences between the curves. Univariate and multivariate Cox regression analyses were applied to evaluate the prognostic effects of the overall survival (OS) and esophageal cancer-specific survival (CSS). SPSS 25 (IBM Corp., Armonk, NY, USA) and GraphPad Prism 8.0 (GraphPad Software, San Diego, CA, USA) were used in later statistical analysis. Two-tailed *P* values less than 0.05 were deemed statistically significant.

## Results

### Demographics

According to the eligibility criteria, 855 elderly EAC patients with DM in stage IVB diagnosed between 2010 and 2015 were included, as the SEER database did not record the information about site-specific metastasis before 2010. The baseline characteristics are displayed in **Table 1**. **Table 1** summarizes the demographic and characteristics of the 855 patients, among which, 733 (85.7%) patients were male; 808 (94.5%) patients were Caucasian. The scales of patients with different patterns of metastasis are summarized in **Table 1**. The most common metastasis site was the liver (40.9%), followed by lung (12.7%), bone (12.0%), and brain (2.8%). The group with multiple metastatic sites had 269 (31.5%) patients. 793 (92.7%) patients had the original tumor located in the lower third of the esophagus. 246 (28.8%) patients had chemotherapy alone, followed by surgery (S) and/or radiotherapy (R) + chemotherapy (C) 189 (22.1%) and surgery and/or radiotherapy 113 (13.2%). Surprisingly, 307 (35.9%) patients (the “N” group in **Tables 1**–**3**) had not received any treatment.

**Table 1:** Characteristics of elderly EAC patients with DM.

Characteristics	≥70
Sex	
Male	733 (85.7%)
Female	122 (14.3%)
Ethnicity	
White	808 (94.5%)
Black	17 (2.0%)
Other	30 (3.5%)
Grade	
I	26 (3.0%)
II	253 (29.6%)
III	423 (49.5%)
IV	10 (1.2%)
Unknown	143 (16.7%)
T stage	
T1	168 (19.6%)
T2	40 (4.7%)
T3	128 (15.0%)
T4	89 (10.4%)
Unknown	430 (50.3%)
N stage	
N0	208 (24.3%)
N1	331 (38.7%)
N2	49 (5.7%)
N3	36 (4.2%)
Unknown	231 (27.0%)
Site of EAC	
Upper	8 (0.9%)
Middle	54 (6.3%)
Lower	793 (92.7%)
Metastasis	
Bone only	103 (12.0%)
Brain only	24 (2.8%)
Liver only	350 (40.9%)
Lung only	109 (12.7%)
Multiple	269 (31.5%)
Treatment	
S or/and R+C	189 (22.1%)
C	246 (28.8%)
S or/and R	113 (13.2%)
N	307 (35.9%)

N, no treatment; S, surgery; R, radiotherapy; C, chemotherapy.

**Table 2:** Results of Univariate and Multivariate analysis using the COX proportional hazards model of the study population.

	Univariate analysis (OS)		Multivariate analysis (OS)		Univariate analysis (CSS)		Multivariate analysis (CSS)	
	HR (95% CI)	P value	HR (95% CI)	P value	HR (95% CI)	P value	HR (95% CI)	P value
Sex								
Male	1	1	1	1	1	1	1	1
Female	1.023 (0.833–1.357)	0.828	0.984 (0.797–1.215)	0.881	1.059 (0.859–1.307)	0.590	1.015 (0.818–1.260)	0.893
Ethnicity								
White	1	1	1	1	1	1	1	1
Black	1.009 (0.594–1.713)	0.973	1.058 (0.619–1.808)	0.837	0.997 (0.576–1.727)	0.992	1.044 (0.599–1.820)	0.879
Other	1.010 (0.678–1.505)	0.962	1.032 (0.689–1.546)	0.879	0.950 (0.621–1.453)	0.812	0.963 (0.627–1.481)	0.865
Grade								
I	1	1	1	1	1	1	1	1
II	1.223 (0.774–1.933)	0.388	1.155 (0.726–1.839)	0.543	1.145 (0.724–1.812)	0.562	1.071 (0.672–1.708)	0.773
III	1.546 (0.985–2.426)	0.058	1.454 (0.917–2.306)	0.112	1.410 (0.898–2.215)	0.136	1.307 (0.822–2.077)	0.258
IV	1.501 (0.702–3.207)	0.295	1.771 (0.805–3.898)	0.155	1.499 (0.702–3.204)	0.296	1.739 (0.789–3.834)	0.170
Unknown	1.215 (0.756–1.954)	0.420	1.068 (0.658–1.734)	0.789	1.180 (0.733–1.899)	0.496	1.020 (0.628–1.658)	0.936
T stage								
T1	1	1	1	1	1	1	1	1
T2	0.570 (0.393–0.826)	0.003	0.557 (0.379–0.820)	0.003	0.574 (0.391–0.841)	0.004	0.549 (0.368–0.818)	0.003
T3	0.663 (0.522–0.843)	0.001	0.831 (0.642–1.077)	0.162	0.652 (0.509–0.836)	0.001	0.798 (0.611–1.043)	0.099
T4	0.870 (0.669–1.133)	0.301	0.989 (0.752–1.301)	0.939	0.898 (0.686–1.176)	0.436	1.004 (0.758–1.328)	0.980
Unknown	0.965 (0.800–1.164)	0.708	0.967 (0.791–1.181)	0.740	0.954 (0.786–1.157)	0.631	0.948 (0.771–1.166)	0.615
N stage								
N0	1	1	1	1	1	1	1	1
N1	0.922 (0.771–1.101)	0.368	1.123 (0.928–1.359)	0.235	0.946 (0.786–1.139)	0.560	1.166 (0.956–1.423)	0.129
N2	0.630 (0.452–0.880)	0.007	0.798 (0.560–1.138)	0.213	0.662 (0.470–0.933)	0.018	0.845 (0.587–1.218)	0.368
N3	0.977 (0.676–1.413)	0.902	1.181 (0.803–1.738)	0.398	1.076 (0.742–1.560)	0.700	1.313 (0.889–1.939)	0.172
Unknown	0.926 (0.750–1.143)	0.472	1.053 (0.842–1.318)	0.650	0.963 (0.774–1.198)	0.736	1.105 (0.876–1.393)	0.399
Site of EAC								
Upper	1	1	1	1	1	1	1	1
Middle	1.312 (0.562–3.064)	0.530	1.375 (0.581–3.250)	0.469	1.235 (0.527–2.892)	0.627	1.334 (0.562–3.167)	0.513
Lower	1.165 (0.521–2.602)	0.710	1.594 (0.705–3.606)	0.263	1.091 (0.488–2.437)	0.833	1.510 (0.667–3.420)	0.323
Site-of metastasis								
Bone only	1	1	1	1	1	1	1	1
Brain only	0.813 (0.501–1.318)	0.400	0.928 (0.565–1.526)	0.770	0.739 (0.439–1.244)	0.255	0.853 (0.501–1.455)	0.561
Liver only	0.920 (0.728–1.162)	0.483	0.746 (0.583–0.954)	0.020	0.932 (0.732–1.187)	0.567	0.769 (0.597–0.992)	0.043
Lung only	0.820 (0.614–1.096)	0.180	0.730 (0.543–0.981)	0.037	0.794 (0.587–1.074)	0.135	0.719 (0.528–0.979)	0.036
Multiple	1.190 (0.936–1.512)	0.156	1.037 (0.811–1.327)	0.770	1.194 (0.932–1.530)	0.160	1.052 (0.816–1.357)	0.695
Treatment								
S or/and R+C	1	1	1	1	1	1	1	1
C	0.990 (0.799–1.228)	0.930	1.014 (0.810–1.268)	0.907	1.032 (0.825–1.290)	0.784	1.036 (0.821–1.306)	0.768
S or/and R	2.123 (1.653–2.728)	0.000	2.232 (1.730–2.880)	0.000	2.235 (1.726–2.894)	0.000	2.345 (1.803–3.050)	0.000
N	4.308 (3.500–5.301)	0.000	4.782 (3.821–5.984)	0.000	4.358 (3.509–5.411)	0.000	4.750 (3.762–5.998)	0.000

N, no treatment; S, surgery; R, radiotherapy; C, chemotherapy.

**Table 3:** Results of Multivariate analysis using the COX proportional hazards model of the different populations.

Site-of metastasis n (%)	Treatment	OS		CSS	
		HR (95% CI)	P value	HR (95% CI)	P value
Bone 103 (12.0%)	S or/and R+C	1	1	1	1
	C	1.503 (0.774–3.036)	0.256	1.557 (0.757–3.205)	0.229
	S or/and R	3.322 (1.601–6.894)	0.001	3.328 (1.579–7.016)	0.002
	N	24.587 (10.183–59.361)	0.000	23.970 (9.760–58.873)	0.000
Brain 24 (2.8%)	S or/and R+C	1	1	1	1
	C				
	S or/and R	123.423 (5.256–2898.474)	0.003	1.000 (0.104–9.586)	1.000
	N	97.716 (0.983–9716.474)	0.051	1.000 (0.007–144.826)	1.000
Liver 350 (40.9%)	S or/and R+C	1	1	1	1
	C	1.099 (0.737–1.639)	0.643	1.074 (0.714–1.614)	0.733
	S or/and R	2.787 (1.700–4.567)	0.000	2.891 (1.755–4.763)	0.000
	N	4.040 (2.734–5.971)	0.000	3.916 (2.629–5.833)	0.000
Lung 109 (12.7%)	S or/and R+C	1	1	1	1
	C	1.111 (0.571–2.160)	0.758	1.116 (0.541–2.300)	0.766
	S or/and R	3.318 (1.342–8.204)	0.009	4.065 (1.582–10.447)	0.004
	N	6.378 (3.138–12.963)	0.000	7.215 (3.365–15.472)	0.000
Multiple 269 (31.5%)	S or/and R+C	1	1	1	1
	C	0.764 (0.514–1.137)	0.185	0.816 (0.543–1.228)	0.330
	S or/and R	2.082 (1.320–3.284)	0.002	2.122 (1.324–3.401)	0.002
	N	5.354 (3.556–8.060)	0.000	5.376 (3.512–8.227)	0.000

OS, overall survival; CSS, cancer-specific survival; N, no treatment; S, surgery; R, radiotherapy; C, chemotherapy.

### Risks Examined for Association With Different Metastasis Sites and Treatments

Multivariate analysis of EAC patients with different DM indicated that the treatment can be the only independent prognostic factor affecting OS and CSS (**Tables 2**, **3**).

As shown in **Table 2**, treatment was associated with OS and CSS. Compared to the S and/or R+C group, the S and/or R group had the poorer OS (HR: 2.232, 95% CI: 1.730–2.880, *p* < 0.001) and the N group showed the worst OS (HR: 4.308, 95% CI: 3.500–5.301, *p* < 0.001). The results of C group and S and/or R+C group were not statistically significant in OS (HR: 1.014, 95% CI: 0.810–1.268, *p* = 0.907). Compared to the bone-only group, liver-only group (HR: 0.746, 95% CI: 0.583–0.954, *p* < 0.05), and lung-only group (HR: 0.730, 95% CI: 0.543–0.981, *p* < 0.05) had better OS. Multiple-site group (HR: 1.037, 95% CI: 0811–1.327, *p* = 0.770) had the worst OS. The similar results were found for CSS. The data in **Table 2** also show that the bone-only group had the worst OS and CSS among the single-organ metastasis population. The multiple-site group had the worst OS and CSS across the population.

As shown in **Table 3**, due to the lack of patients in the brain-only group (only 24 patients), we were unable to draw statistically significant results, so we did not consider this group; however, we found similar results in the other four groups (across the population). The results showed that compared to S and/or R+C group, the S and/or R group had the worse OS and CSS, and the N group showed the worst OS and CSS. The results of C group and S and/or R+C group were not statistically significant in OS and CSS.

In **Table 4**, data show that the MST in OS were 3, 5, 4, 3, and 2 months and the MST values in CSS were 4, 6, 4, 4, and 3 months in the bone-only, brain-only, lung-only, liver-only, and multiple-site groups, respectively. The 6-month survival rate (SR), 1-year SR, and 3-year SR were lowest in the multiple-site group than the other groups (6-month SR: 27.1% in OS, 29.9% in CSS, 1-year SR: 10.7% in OS, 12.0% in CSS, 3-year SR: 2.5% in OS, 2.8% in CSS). These results show that patients with multiple-site metastases had the lowest 6-month SR and 1-year SR among all populations, and patients with bone-only metastasis had the worst 6-month SR and 1-year SR among those with single-organ metastasis.

**Table 4:** Prognostic analysis in different population.

Site of Metastasis		OS	CSS
Bone	MST (month)	3	4
	6-month SR (%)	35.6	38.2
	1-year SR (%)	20.0	21.5
	3-year SR (%)	1.9	2.7
Brain	MST (month)	5	6
	6-month SR (%)	36.4	40.6
	1-year SR (%)	27.3	30.4
	3-year SR (%)	0	0
Liver	MST (month)	4	4
	6-month SR (%)	36.5	38.6
	1-year SR (%)	21.1	23.6
	3-year SR (%)	2.1	2.5
Lung	MST (month)	3	4
	6-month SR (%)	43.5	45.6
	1-year SR (%)	23.9	28.2
	3-year SR (%)	4.9	8.4
Multiple	MST (month)	2	3
	6-month SR (%)	27.1	29.9
	1-year SR (%)	10.7	12.0
	3-year SR (%)	2.5	2.8
Total	MST (month)	3	4
	6-month SR (%)	34.3	36.8
	1-year SR (%)	18.1	20.5
	3-year SR (%)	2.4	3.2

OS, overall survival; CSS, cancer-specific survival; MST, median survival time; yrs, years; SR, survival rate.

**Figure 2:**
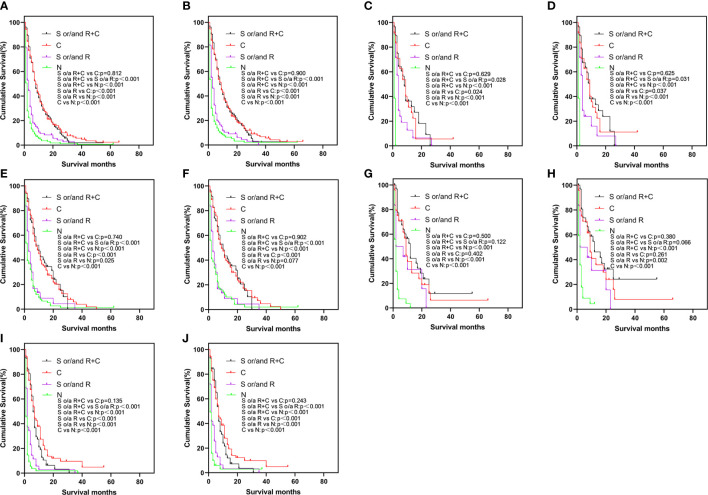
Kaplan-Meier curve of OS **(A)** and CSS **(B)** by different treatments in the total study population, OS **(C)** and CSS **(D)** in bone-only group, OS **(E)** and CSS **(F)** in liver-only group, OS **(G)** and CSS **(H)** in lung-only group, OS **(I)** and CSS **(J)** in the multiple-site group. OS, overall survival; CSS, cancer-specific survival.

To elucidate the relationship about these treatment modalities for prognosis, a Kaplan-Meier survival analysis was undertaken (**Figure 2**). In **Figures 2A, B**, in the elderly patient population, the prognosis of different treatment modalities for elderly patients varied greatly. Regardless of whether the patient was treated with surgery or radiotherapy, the prognosis of elderly patients treated with chemotherapy was better than that of treatment without chemotherapy (*p* < 0.001). The prognosis of surgery and/or radiotherapy was better than that of untreated patients (OS and CSS: *p* < 0.001). However, the prognosis of surgery and/or radiotherapy + chemotherapy was not statistically different from chemotherapy alone (OS: *p *= 0.812, CSS: *p* = 0.900). The similar results were also found in bone-only, liver-only, and multiple-site groups (**Figures 2C–F, I, J**). In **Figures 2G, H**, the prognosis results of surgery and/or radiotherapy + chemotherapy, surgery and/or radiotherapy, and chemotherapy alone were not statistically significant in the lung-only group.

## Discussion

In our present study, the metastasis patterns and prognosis of EAC elderly patients with DM in stage IVB were investigated. Our results indicated that the most common site of metastasis was liver, followed by lung, bone, and brain among the single-organ metastasis population. In addition, treatment was an independent prognostic factor affecting OS and CSS; because the treatment of multiple-site metastasis was limited, the prognosis of patients with multiple-site metastasis was even worse. Chemotherapy played an essential role in EAC elderly patients with DM in stage IVB. Regardless of whether the patient was treated with surgery or radiotherapy, the prognosis of elderly patients who were treated with chemotherapy was better than that without.

According to autopsy results, the lung and liver were the most common metastatic organs in patients with EC, with 31% and 23% of patients therein, respectively (17). Some studies have shown that the liver and lung were the most common metastatic organs in patients with EC (18). Bone was also a common organ for DM (19, 20). Bone metastasis was the third most common site of metastasis in patients with EC (18, 21). Other studies have reported that the most common site for metastasis was the liver, followed by lung, bone, and brain (22–25). Moreover, there are limited studies on the effect of the DM on survival in metastatic EC. San-Gang Wu et al. found that the site of metastasis showed an effect on survival in metastatic EC and bone metastasis had the poorest OS, which was greatest for distant lymph node metastases (26). The study by Jin Zhang et al. included EC patients with bone metastasis and showed the prognostic factors for bone metastases patient survival in EC (27). However, Tanaka et al. found that there was no significant difference in median survival among different sites of DM, which included bone, liver, and lung (28). The mechanisms of bone metastasis leading to lower survival rate of metastatic EC than other sites remain unclear. Overproduced parathyroid hormone-related peptide (PTHrP) is usually caused by osteolytic bone metastasis (29). Hypercalcemia and leukocytosis are associated with bone metastasis in EC, which may induce rapid disease progression (30–32).

EC is often manifest as transmural invasion with far advanced and early metastatic spread at diagnosis. EC patients with distant organ metastasis are classified as stage IVB in the TNM classification and have not been treated with curative intent (in general). These patients often have been considered as candidates for palliative therapy, such as photodynamic therapy, stent placement, and palliative chemotherapy or radiotherapy (33–37). Thus, the prognosis of these patients relies on the degree of spread and is, in general, extremely poor, usually with an MST of less than 6 months.

In a sense, radiotherapy and chemotherapy and surgery are in fact palliative treatments for EAC patients with DM, but our results have shown the prognosis of no treatment < surgery and/or radiotherapy < surgery and/or radiotherapy + chemotherapy (**Figure 2**). Furthermore, chemotherapy had shown obvious benefits to the prognosis: according to our results, whether elderly patients received chemotherapy, radiotherapy, or surgery had an important effect on the prognosis. Since there were only 27 patients who had undergone surgery, thus, the results were mainly about the effects of radiotherapy and chemotherapy on the prognosis of EAC elderly patients with DM. Our results suggested that active treatment can significantly improve the prognosis of patients, and chemotherapy had played a more important role. This study proved that radiotherapy (such as stereotactic body radiotherapy and radio-frequency ablation) and chemotherapy (very necessary to treat DM patients) can help extend the survival time. The results in the lung-only group were not statistically significant in terms of difference between surgery and/or radiotherapy + chemotherapy, chemotherapy alone and surgery and/or radiotherapy. However, the prognosis of no treatment < surgery and/or radiotherapy < surgery or/and radiotherapy + chemotherapy was similar in other groups.

Although multimodal therapy has been considered as an effective treatment in locally advanced primary EC patients, its role in treating patients with DM remains poorly defined (9, 33). There is little research into the application of multimodal therapy in patients with DM (38–41). In our study, there was no difference in survival between patients treated with surgery or/and radiotherapy + chemotherapy and those treated with chemotherapy alone. Surgery and radiotherapy are not considered beneficial as the treatment modality for these patients with chemotherapy: chemotherapy can be deemed to be the primary mode of treatment.

Although all treatment modalities are considered as palliative treatment for patients with DM, chemotherapy is a systemic approach, which can treat other metastatic organs of EC patients, compared to radiotherapy, the local therapy modality and the results reflected this finding.

Our results showed that 307 (35.9%) patients did not have any treatment. The low percentage of active treatment limited the OS and CSS of elderly patients. The study by Basile Njei et al. showed that no surgery and no radiotherapy were independent negative prognostic factors that affect the prognosis of patients with EC (42).

We think the reasons for the poor prognosis of EAC elderly patients with DM in stage IVB were also mainly determined by low percentage of active treatment. The reasons for that include the doctors’ opinions and patient-related factors.

1. Doctor’s point of view. Due to the presence of more complications in elderly patients, strict surgical indications limit surgical opportunities for elderly patients. Considering age-related health conditions, operative adverse events, and mortality, doctors tend to treat conservatively. Safe implementation of beneficial treatments at standard doses to improve survival and whether treatment-related side effects may influence the quality of life of patients become two conflicting aspects that clinicians need to consider.
2. Factors related to patients. Elderly patients are more likely to consider not taking active treatment. There are also many studies on malignant tumors that the reasons for patients’ refusal to be treated may be associated with increased delivery of suboptimal therapy, decreased referral to specialists and increased patient refusal of therapy (43–46). The reasons that lead elderly patients to be treated conservatively also include the existence of comorbidities that affect the patients’ drug absorption and/or metabolism (46, 47).

In addition, senescent cells can also secrete many growth factors to promote the growth of tumor cells. Age, the number of complications, the presence of tumors, and deep vein thrombosis associated with aging can significantly increase the risk of death (48, 49).

About 65% of cancer patients are over 65 years of age, with the increase in life expectancy, this may rise to 70% in the future (50), however, among those patients who participated in cancer-related clinical trials, only 40% were over 65 years of age, and no more than 10% were over 75 (51), therefore, clinical trials of cancer treatments need to focus on elderly patients.

The study has certain limitations: this is a retrospective study and many factors contributing to the possible poor prognosis for this patient group are missing. The study is generalized to the American population, but lacked inclusion of cases among ethnic minorities, thus the results can not represent the global population. Compared with the most common sites, such as liver, lung, and bone, brain metastasis is rare, which makes the survival results of patients with brain metastasis subject to statistical bias. Different patterns of multiple metastatic sites were not differentiated, such as bone + brain, bone + liver, bone + lung, bone + brain + liver, etc. Immunotherapy and targeted therapy were not taken into consideration. Due to the flaws in the database, we cannot know the detailed information of the treatment modalities such as surgery, radiotherapy, and chemotherapy, which may affect the results of the study. On the other hand, there are no relevant reports on elderly EC patients with DM, thus, the study on metastasis and prognosis of such patients shows an important significance.

In conclusion, metastasis patterns and survival outcomes of EAC patients with DM in stage IVB were studied in elderly patients. Elderly patients with multiple-site metastasis had the worst OS and CSS. Patients with bone-only metastasis had the worst OS and CSS among single-organ metastasis populations. Patients with active treatments had better CSS and OS. Chemotherapy was beneficial to these patients, however, over 35.9% patients more than 70 years of age did not take any anti-cancer treatment. These elderly patients had highest rate of cancer-specific deaths among the study population.

## Data Availability Statement

The raw data supporting the conclusions of this article will be made available by the authors, without undue reservation.

## Ethics Statement

The studies involving human participants were reviewed and approved by the ethics committee of the West China Hospital. Written informed consent for participation was not required for this study in accordance with the national legislation and the institutional requirements.

## Author Contributions

GQ contributed the idea. HZ contributed the design of the study. FW contributed the calculation. YZ contributed the proofread. ZW contributed error correction. YW contributed the idea. All authors contributed to the article and approved the submitted version.

## Funding

This study was supported by the National Key Research Project Fund of China (grant no. 2017YFC0113502) and the Key Research Project of Sichuan Province (grant no. 2020YFS0249).

## Conflict of Interest

The authors declare that the research was conducted in the absence of any commercial or financial relationships that could be construed as a potential conflict of interest.
